# Synthesis, Characterization, and Evaluation of Genistein-Loaded Zein/Carboxymethyl Chitosan Nanoparticles with Improved Water Dispersibility, Enhanced Antioxidant Activity, and Controlled Release Property

**DOI:** 10.3390/foods9111604

**Published:** 2020-11-04

**Authors:** Yu Xiao, Chi-Tang Ho, Yulian Chen, Yuanliang Wang, Zihao Wei, Mingsheng Dong, Qingrong Huang

**Affiliations:** 1 College of Food Science and Technology, Hunan Agricultural University, Changsha 410128, China; xiaoyu@hunau.edu.cn (Y.X.); wangyuanliang@hunau.edu.cn (Y.W.); 2 College of Food Science and Technology, Nanjing Agricultural University, Nanjing 210095, China; 3 Department of Food Science, Rutgers University, 65 Dudley Road, New Brunswick, NJ 08901, USA; ctho@sebs.rutgers.edu (C.-T.H.); weizihao@ouc.edu.cn (Z.W.); 4 College of Animal Science and Technology, Hunan Agricultural University, Changsha 410128, China; yulianchen@stu.hunau.edu.cn

**Keywords:** genistein, zein, carboxymethyl chitosan, nanoparticles, water dispersibility, stability

## Abstract

Genistein is one of major isoflavones derived from soybean products and it is believed to have beneficial effects on human health. However, its low water-solubility and poor oral bioavailability severely hamper its use as a functional food ingredient or for pharmaceutical industry. In this study, zein and zein/carboxymethyl chitosan (CMCS) nanoparticles were prepared to encapsulate genistein using a combined liquid–liquid phase separation method. The physicochemical properties of fabricated nanoparticles were characterized by dynamic light scattering (DLS), atomic force microscopy (AFM), and Fourier transform infrared spectroscopy (FTIR). The results demonstrated that genistein encapsulated with zein nanoparticles significantly improved its water dispersibility, antioxidant activity in the aqueous phase, and photostability against UV light. Moreover, genistein encapsulated in zein nanoparticles showed a sustained release property. Furthermore, it was found that encapsulation efficiency of genistein was significantly enhanced after CMCS coating, and this effect was more pronounced after the complex nanoparticles cross-linked with calcium ions when compared with the use of zein as a single encapsulant. In addition, compared to zein nanoparticles without biopolymer coating, CMCS coating significantly enhanced the thermal and storage stability of the formed nanoparticles, and delayed the release of genistein. A schematic diagram of zein and zein/carboxymethyl chitosan (CMCS) nanoparticles formation mechanism for encapsulation of genistein was proposed. According to the results of the current study, it could be concluded that encapsulation of genistein in zein/CMCS nanoparticles is a promising approach to improve its water dispersibility, antioxidant activity, photostability against UV light and provide controlled release for food/pharmaceutical applications.

## 1. Introduction

**Figure 1:**
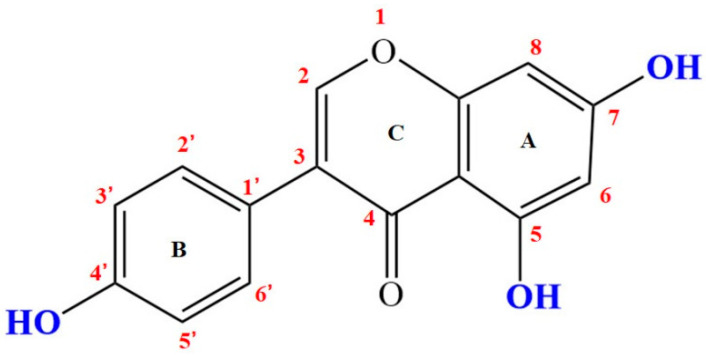
The chemical structure of genistein (4′, 5, 7-trihydroxyisoflavone, CAS NO. 446-72-0).

In recent years, the role of diet on human health has received increased attention. Previous studies have demonstrated that a high intake of soy products in Asian countries (e.g., China, Japan, India, Korea) contribute to their lower incidences of chronic diseases, such as obesity, prostate/ breast cancers, and cardiovascular disease, especially when compared to western populations [1]. There is a growing evidence body that genistein (4′,5,7-trihydroxyisoflavone) (Figure 1), one of the primary isoflavone aglycones in soybean products (especially for fermented soybean products) [2,3], contributes to many of these health benefit effects. For example, it is thought to lower the incidence of prostate, breast, and colon cancer, reduce the risk of cardiovascular disease, improve bone health, and attenuate postmenopausal symptoms [4,5,6]. For these reasons, genistein has become a popular nutraceutical supplement and received growing interest around the world in the last decade. It is commonly used as a nutraceutical ingredient in the food industry or as a drug in pharmaceutical industry. However, the introduction of nutraceuticals into food products has many technological challenges due to the highly unsaturated structure of nutraceuticals, which are sensitive to light, heat, and oxidation, thereby reducing their potential health benefits [7]. Furthermore, genistein shows low aqueous solubility and poor oral bioavailability, which limits its applicability as a nutraceutical ingredient in the food industry. Moreover, genistein is rapidly metabolized after oral administration, severely affecting the absorption of this bioactive compound [8,9]. Therefore, there is an urgent need for an economical and feasible delivery system to enhance the solubility and dissolution rates, and bioavailability of genistein while maintaining its chemical stability, and controlling its release in the food product as well.

In the food and pharmaceutical industries, encapsulation of poor water-soluble bioactive compounds has gained great attention. This novel approach is able to increase the water solubility and stability of bioactive compounds, allowing them to maintain health promoting effects during processing and storage [10]. In addition, previous studies also reported that nutraceuticals could achieve targeted delivery and controlled release after encapsulation, thereby enhancing their biological activity [11]. Recently, some studies have been reported to improve the water solubility and bioavailability of genistein by encapsulation [12,13]. For example, cyclodextrins and their derivates, cyclodextrin/hydrophilic polymer mixtures, surfactants, hyperbranched polyester, and emulsions, were used as potential carriers to enhance isoflavones’ solubility and bioavailability [9,14]. However, many shortcomings still exist in these strategies, which inhibits further application of genistein as a supplement as well as industrial production. For instance, nanoencapsulation of genistein was recently developed using a synthetic compound star-shaped copolymermannitol-functionalized poly(d,L-lactide-co-glycolide) d-α-tocopheryl polyethylene glycol 1000 succinate (PLGA-TPGS) by Wu et al. [12]. In their study, although controlled release and superior antitumor activity were achieved, toxic solvents (i.e., acetone) and synthetic compounds were used concurrently, which had potential side effects on the final product as a result of residues of the toxic solvent and non-biodegradable compound [12].

In recent years, proteins used as potential encapsulants for bioactive components have received increased attention [15,16]. Proteins are metabolizable, biodegradable, biocompatible, and non-toxic, thus making them excellent building materials in food applications. It has been reported that some proteins produce nanoparticles, e.g., gluten, human serum albumin, gliadin, and gelatin [16,17,18]. Zein, the prolamin protein from corn, consists of one-fourth hydrophilic and three-fourths lipophilic amino acid residues, which possesses promising advantages in food applications [19]. The water insoluble but alcohol-soluble characteristics of zein makes it favorable for encapsulating and delivering bioactive compounds that have low water solubility but are soluble in ethanol. It has been documented that zein nanoparticles could be formed, simultaneously encapsulating the alcohol-soluble components (e.g., fish oil, VD3, essential oil, curcumin, resveratrol) in one step via simply enhancing the solvent polarity, which is conducive to industrial scale production [15,16,19,20]. Although zein-based nanoparticles showed unique advantages, inadequate encapsulation of bioactive compounds and poor nanoparticles stability were also found to hamper its application [21]. In order to overcome these issues, previous investigators have used polysaccharide coating on zein nanoparticles. As a major class of biopolymers, polysaccharides are widely used in food industry. Carboxymethyl chitosan (CMCS), a water-soluble derivative of chitosan, has been reported to coat protein nanoparticles for drugs/nutraceuticals delivery due to its bio-adhesive promoting effect and controllable self-aggregation characteristic [17,22,23]. CMCS coated protein nanoparticles have been demonstrated to be a very useful delivery system to enhance the encapsulation efficiency and improve release profiles of water insoluble nutrients, such as propolis, curcumin, and VD3 [17,19,22,24]. In addition, the complexed nanoparticles could enhance the protective effects of encapsulated compounds against light and heat-induced degradation, probably due to a more compact structure has been formed [17].

The effect of encapsulation with protein nanoparticles on the release property, antioxidant activity, and water dispersibility of VD3, propolis, curcumin, resveratrol, and fish oil has been studied previously [20,24,25,26]. However, to the best of our knowledge, no information is available on the effect of encapsulation with zein nanoparticles on the antioxidant activity and water dispersibility of genistein. Therefore, in this study, genistein was firstly encapsulated into zein nanoparticles and then we investigated the effects of encapsulation on water dispersibility, antioxidant activity, stability, and release behavior of genistein. The formed zein nanoparticles was further coated with CMCS and cross-linked with calcium ions to investigate the effect of coating on the thermal and storage stability of zein-nanoparticles, encapsulation efficiency, and photo-stability of genistein. The obtained nanoparticles were characterized for particulate characteristics, such as particle size distribution, polydispersibility, loading efficiency, and encapsulation efficiency. Dynamic light scattering (DLS), Fourier transform infrared spectrometry (FTIR), zeta potential analysis, and atomic force microscopy (AFM) were used to study the possible interactions during the encapsulation process, as well as to investigate the morphology of the obtained particles. The obtained results of the present study are valuable for assessing the potential of genistein-loaded biopolymeric particles supplemented in food products and beverages without affecting product quality.

## 2. Materials and Methods

### 2.1. Materials

Zein (with 98% purity) was obtained from Japan Wako Pure Chemical Industries. The compounds genistein (with 98% purity), 2,4,6-tris(2-pyridyl)-S-triazine (TPTZ), and 2, 2-azinobis (3-ethylbenzothiazoline-6-sulfonic acid) diammonium salt (ABTS) were purchased from Sigma-Aldrich Chemical Co. Ltd. (St. Louis, MO, USA). CMCS (a deacetylation degree of 99%) was obtained from Nantong Xing Cheng Biological Product Inc. (Nantong, China). All other chemicals were of analytical grade and purchased from Fisher Scientific (Waltham, MA, USA).

### 2.2. Fabrication of Genistein-Load Zein Nanoparticles

The zein nanoparticles were fabricated following a phase separation method according to previous studies with some modifications [17,19]. The optimum condition to form stable nanoparticles was conducted by preliminary trials. Briefly, zein was dissolved in an 80% aqueous ethanol binary solvent to form a zein stock solution with 6 mg/mL. Genistein (1.2 mg/mL) was also prepared in an 80% aqueous ethanol solvent as a stock solution. A weighed amount of CMCS powder was dissolved in water to obtain the solution with 6 mg/mL. Next, 1 mL of genistein solution was added dropwise to 2 mL zein solution under continuous stirring in a 30 mL glass vial for 60 min. After that, the mixture was added dropwise into 5 mL CMCS solution with magnetic stirring for 30 min at 500 rpm. Then, 1 mL of 0.4 mg/mL calcium chloride was added dropwise into the above mixture. The resulting mixture was then continuously stirred for another 30 min at 500 rpm. These dispersions were centrifuged for 10 min at 987× *g* to separate any larger aggregates if formed. The obtained dispersions were subsequently lyophilized for 48 h to obtain solidified genistein encapsulated zein nanoparticles (G-ZCMC/C). The other two kinds of nanoparticles without CMCS and calcium ion (G-Z), as well as without calcium ion (G-ZCMC), were also fabricated for comparison. In addition, native genistein (1 mL of 1.2 mg/mL genistein solution was added dropwise to 2 mL of 80% aqueous ethanol and then dispersed into 6 mL of deionized water) was also prepared. Appearances of native genistein, G-Z, G-ZCMC, and G-ZCMC/C in H_2_O after 12 h storage at room temperature were recoded, and the content of the genistein in the dispersion was analyzed by high performance liquid chromatography (HPLC). The absorbance of the studied samples at 500 nm was measured to compare their turbidity. A UV-vis spectrometer (Cary 60 UV-vis, Agilent Technologies) was used to determine the absorbance spectra of each obtained sample. The scanning range was from 200 to 800 nm. 

### 2.3. Analysis of Particle Size Distribution, Polydispersity Index (PDI) and Zeta-Potential 

The particle sizes and PDI of freshly produced samples were determined using dynamic light scattering (DLS, BIC 90 Plus particle size analyzer, Brookhaven Instrument Corp., Holtsville, NY, USA) with a fixed scattering angle of 90°. The refraction index was set at 1.333 (1.330 for pure water and 1.355 for pure ethanol). A Malven Zetasizer Nano-ZS 90 (Malvern Instruments Ltd., Works, UK) was carried out to determine the zeta-potential. All measurements of samples were performed in triplicates and conducted at 25.0 °C.

### 2.4. Measurement of Loading Efficiency (LE) and Encapsulation Efficiency (EE) of Genistein

LE and EE was determined based on the method of Luo et al. [19] with some modifications. In brief, 10 mg of lyophilized sample was rinsed with ethyl acetate (2 mL) three times to wash off free genistein which performed on No. 1 Whatman filter paper. After washing, the nanoparticle samples were dried by vacuum and then extracted by 80% aqueous ethanol (*v*/*v*) in an ultrasonication instrument. The content of genistein was determined by HPLC after the samples filtered with 0.2 μm membrane. EE and LE were calculated in light of the following equations: EE = (encapsulated genistein/genistein input) × 100%; LE = (encapsulated genistein/weight of nanoparticles) × 100%.

### 2.5. Morphological Studies by Atomic Force Microscopy (AFM)

Morphology of the nanoparticles was studied using a NanoScope IIIA Multimode AFM equipped with a silicon-etched RTESP7 cantilever (Veeco Nanoprobe, Santa Barbara, CA, USA). The freshly prepared Z-G, Z-GCMC, and Z-GCMC/C dispersions were diluted in water and then deposited onto freshly cleaved mica. Samples were dried under a stream of nitrogen after 1 h of absorption, and then the samples were mounted on scanner tubes. Spring constant of 40 N/m for the silicon tip was used in this study. Fixed scanning rate of 0.997 Hz was used to simultaneously capture high-mode images with a resolution of 512 × 512 pixels and a size of 5 μm × 5 μm. All the height photographs are showed after the first-order two-dimensional (2D) flattening.

### 2.6. Solid-State Characterization by Fourier Transform Infrared Spectroscopy (FTIR)

The lyophilized powders were pressed into a mold to make a tablet. The pure ingredients (zein, CMCS and genistein) and nanoparticulate genistein formulations were investigated by a Thermo Nicolet Nexus 670 FTIR spectrophotometer (Thermo Fisher Scientific Inc., Walsham, MA, USA). Absorption spectra of samples were obtained at 4000 to 500 cm^−1^ wavenumbers with a resolution of 4 cm^−1^ and the air was used as the background, and then smoothing the original FTIR spectra with smoothing points using OMNIC 7.2 software [17]. All experiments were conducted at 25 °C and performed in triplicate.

### 2.7. Analysis of Stability to Environmental Conditions

#### 2.7.1. Stability to Thermal Processing

Thermal stability was determined according to Joye et al. [16]. Briefly, freshly fabricated nanoparticle dispersions were put in different glass test tubes and incubated for 30 min in water baths with temperatures ranging from 30 to 90 °C. After cooling down to 23 °C and stored for 24 h, the particle size, PDI, and zeta potential of the dispersions was analyzed as described in Section 2.3. The thermal stability of produced nanoparticles were also studied that heated for different time (1–8 h) at the same heated temperatures (65 °C). 

#### 2.7.2. Stability to Storage 

Storage stability was evaluated using the method as reported by Huang et al. [27]. In brief, freshly fabricated nanoparticles were brought to different glass test tubes, and then stored at 4 °C (refrigeration temperature) and 25 °C (room temperature) for 60 days, respectively. An aliquot of the samples was withdrawn during different time intervals to evaluate the change of particle size, PDI, and zeta potential as described in Section 2.3.

### 2.8. Evaluation of Antioxidant Activity of Genistein-Load Zein Nanoparticles

#### 2.8.1. Estimation of ABTS Radical Cation Scavenging Activity

The antioxidant activity of genistein-loaded nanoparticles was assessed by the ABTS radical cation (ABTS^+^) scavenging activity according to the method reported by Chang et al. [28] and Xiao et al. [29]. In brief, ABTS^+^ stock solution was obtained by mixing 2.45 mM K_2_S_2_O_8_ aqueous solution with 7 mM ABTS aqueous solution and stored in the dark at 25 °C for 16 h. The ABTS working solution was prepared after the stock solution diluted with deionized water to obtain absorbance at 734 nm of 0.70 (±0.02). Then, about 320 μL of each sample of nanoparticles was mixed with 4 mL ABTS^+^ working solution and incubated in dark for 6 min, and subsequently recorded the absorbance at 734 nm. ABTS^+^ scavenging activity was evaluated in light of the following equation: ABTS^+^ scavenging activity (%) = [(*A_control_* − *A_sample_*)/*A_control_*] × 100. Where *A_control_* was the absorbance for the blank group without the sample and *A_sample_* was the absorbance for the studied sample group. Two controls were carried out according to the above method for comparison, namely, genistein pre-dissolved in an 80% aqueous ethanol solvent (1.2 mg/mL) and then diluted with deionized water (Control 1) and a 80% aqueous ethanol solvent (Control 2) to the proper concentrations (5–50 μg/mL).

#### 2.8.2. Assay of Ferric Reducing Antioxidant Power (FRAP)

Antioxidant activity of the genistein-loaded nanoparticles was also assayed by FRAP method as described by Tan et al. [30]. Fresh FRAP reagent contained 100 mL of 0.3 M acetate buffer (pH 3.6), 10 mL of 20 mM ferric chloride, and 10 mL of 10 mM TPTZ (dissolved in 40 mM hydrochloric acid). Further, 1 mL of each sample of nanoparticle dispersions were mixed with 5 mL FRAP reagent and incubated in the dark for 20 min at 37 °C. The absorbance at 593 nm was recorded against a blank. Different concentrations of ferrous sulphate (100–1600 µM) standard solutions were prepared to plot the calibration curve. The final results of FRAP were presented as µM Fe (II). A greater of FRAP value is indication of a stronger antioxidant activity. Two controls which described in the Section 2.8.1 were also conducted in parallel for comparison.

### 2.9. In Vitro Release Profile of Genistein from Nanoparticles

The in vitro releases profile of genistein from G-Z, G-ZCMC, and G-ZCMC/C nanoparticles were performed by using dialysis membrane method [31,32]. Briefly, genistein-loaded nanoparticles were put in the dialysis membrane with molecular cutoff of 10–14 kDa. The sealed dialysis membrane was put in the release medium (200 mL, 50% ethanol) at 37.5 °C and stirred at a speed of 50 rpm. An aliquot of the release sample was withdrawn during the different incubation time intervals. After that, same volume of fresh medium was immediately put into the release medium to maintain the sink conditions. The genistein content was determined by high-performance liquid chromatography (HPLC).

### 2.10. Photostability Test

The photostability of encapsulated genistein and native genistein was estimated according to the report of Xiao et al. [17]. Freshly fabricated nanoparticle dispersions were conducted to stability tests. The control was prepared by dissolving native genistein into an 80% aqueous ethanol and subsequently dispersing it into water, with the final concentration comparable to that of the genistein in the nanoparticles. Samples were transferred into transparent glass vials which irradiated to UV light bulbs (4 W, 365 nm) in a light-proof cabinet for up to 72 h. Further, 400 µL of samples were withdrawn at designated time intervals and then extracted and analyzed by HPLC. The unchanged genistein (%) was plotted against different treatment time.

### 2.11. Analysis of Genistein Content via HPLC

The mobile phase used deionized water as solvent A and acetonitrile as solvent B, and the solvent elution speed was 0.8 mL/min. The column oven temperature kept at 25 °C. Next, 10 µL of the test sample solution was injected into the HPLC system. The program of gradient elution was carried out as follows: starting with 10% B to 35% B in 1 min, then 35–40% B for over 1 min, 40–75% B for 12 min, 75–30% B for 1 min, and finally 30–10% B for 1 min. Samples were detected at 260 nm and quantified based on the standard calibration curve using different concentrations of genistein (0.5–100 μg/mL). The limit of detection (LOD) and the limit of quantification (LOQ) of the method for determination of genistein were 0.02 and 0.07 μg/mL, respectively. The calibration equation is *y* = 1.3622*x* − 0.4411 (*R*^2^ = 0.9992), where y is the peak area and x is the concentration of genistein.

### 2.12. Statistical Analysis

All measurements were carried out in triplicates. Experimental results were expressed as mean ± standard deviation (SD) (*n* = 3). One-way ANOVA with Duncan’s multiple range tests by using SPSS 17.0 software (SPSS Inc., Chicago, IL, USA) were performed to evaluate the significant differences (*p* < 0.05) among different samples.

## 3. Results and Discussion

### 3.1. Physicochemical Characterization of Genistein-Zein/CMCS Nanoparticles

**Figure 2:**
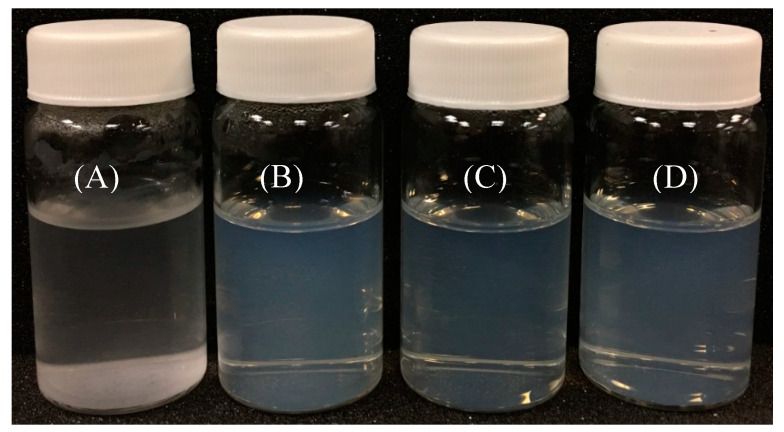
Appearance of native genistein (**A**), G-Z (**B**), G-ZCMC (**C**), and G-ZCMC/C (**D**) nanoparticles in distilled water after 12 h of storage.

**Figure 3:**
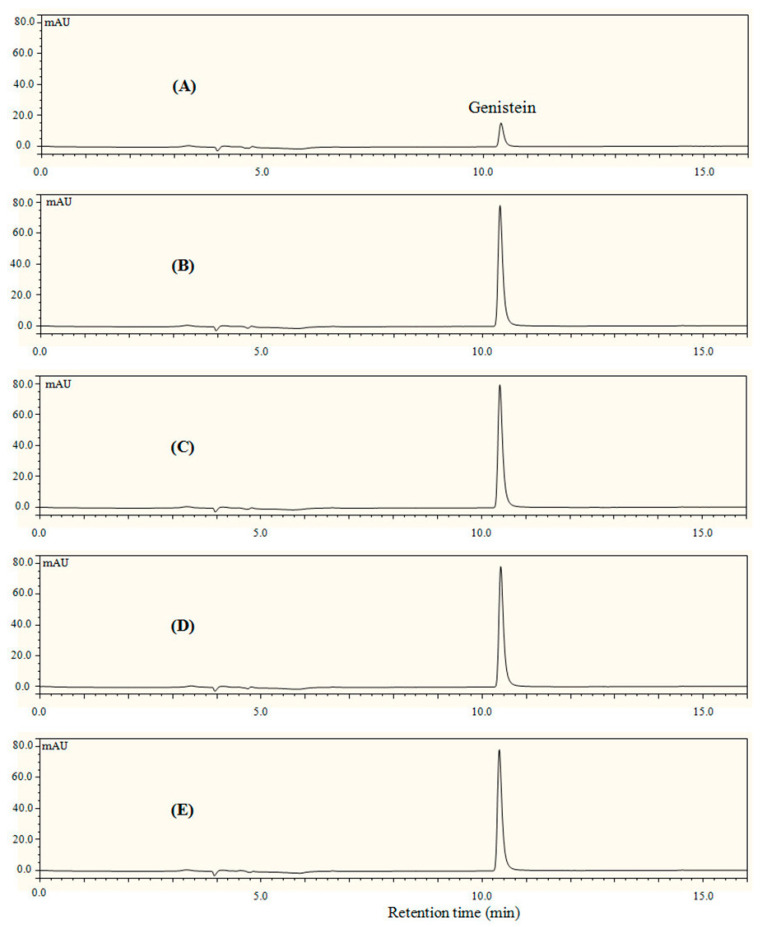
HPLC chromatograms of genistein before (**A**) and after encapsulation (**B**–**D**). Free genistein dissolved in 80% ethanol ((**E**), positive control). (**B**) G-Z; (**C**) G-ZCMC; (**D**) G-ZCMC/C. The samples stand for 24 h when freshly prepared, and then 200 uL of the sample was added with 1 mL 80% ethanol for HPLC analysis.

**Figure 4:**
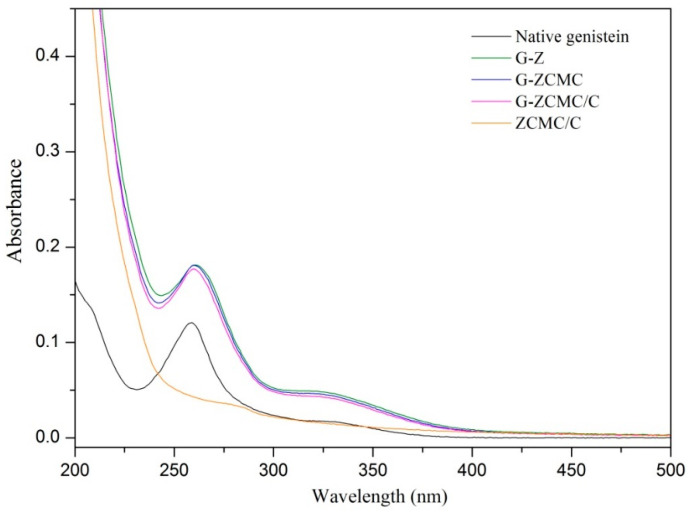
The UV-vis absorption spectra of genistein before and after encapsulation. ZCMC/C: the nanoparticles do not contain genistein.

Native genistein is a water-insoluble compound, but dissolves in organic solvent like ethanol. In the present study, composite colloidal dispersions were fabricated by co-precipitating 80% aqueous ethanol solutions of genistein and zein in water containing CMCS as stabilizer. The digital photos of native genistein, G-Z, G-ZCMC, and G-ZCMC/C are shown in Figure 2. Bluish turbid solution of G-Z, G-ZCMC, and G-ZCMC/C complex formation was found and no precipitate for the three dispersions was observed during the whole storage process (12 h) along with the dispersibility study. It is apparent that the genistein was well dispersed in all the three complex formations. However, it can be seen that the precipitation process started at 4 h for control group (native genistein), and most of the genistein precipitated after storage for 12 h. The phenomenon observed was due to the hydrophobic interaction between the genistein and bulk water solution. In addition, it was also found that only the native genistein suspension yielded precipitation after the dispersions centrifuged at 2743× *g* for 10 min, while genistein in G-Z, G-ZCMC, and G-ZCMC/C maintained the uniform dispersion state, indicating that the water dispersibility of genistein was greatly improved after being encapsulated in zein nanoparticles. The HPLC result of the dispersed genistein in the prepared four genistein samples is shown in Figure 3. It was noted that the pure genistein dissolved in 80% ethanol (Figure 3E) exhibited a sharp peak at the retention time of 10.6 min. The genistein from G-Z, G-ZCMC, and G-ZCMC/C had the major peak with the same retention time and peak area compared with the pure genistein dissolved in 80% ethanol. However, the genistein content in the genistein suspension (stock genistein dispersed in water, Figure 3A) was much lower compared with G-Z, G-ZCMC, and G-ZCMC/C dispersions. These results further indicated that genistein were 100% dispersed in the three zein colloidal complex but were very weakly dispersed in bulk water. The UV-vis absorption spectra of genistein before and after encapsulation also proved that the zein colloidal complex significantly enhanced the dispersibility of genistein in am aqueous condition (Figure 4). Furthermore, from the results of HPLC and UV-vis, we speculated that the chemical structure of genistein might not have changed after it was complexed with zein or CMCS/Ca under the studied conditions. However, more investigations (e.g., MS, NMR, etc.) are required to fully confirm this speculation.

**Figure 5:**
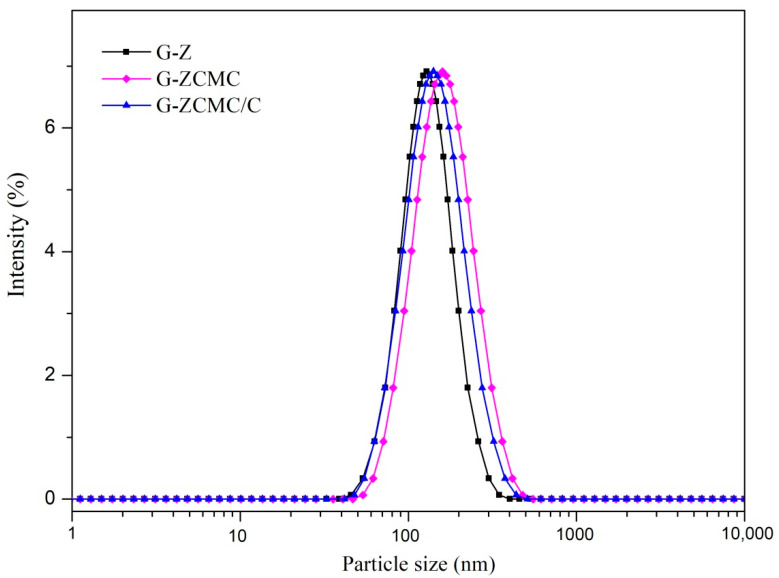
Size distribution of different genistein-loaded zein nanoparticles.

The polydispersity index (PDI) and particle size of G-Z, G-ZCMC, and G-ZCMC/C are presented in Table 1, and the particle distributions are revealed in Figure 5. It was found that the average particle size of G-Z is 128.33 ± 3.09 nm in the absence of CMCS. After adding CMCS, the average particle size increased to 159.20 ± 3.18 nm. The increase of particle size indicated that CMCS was involved in particle formation. However, the average particle size then decreased to 140.37 ± 3.39 nm when zein/CMCS nanoparticles cross-linked with calcium ions. The particle size of all three genistein-loaded zein nanoparticles are much lower than that of previous studies who reported that genistein-loaded onto lipid nanoparticles or star-shaped mannitol-core PLGA-TPGS nanoparticles [12,33]. The PDI values of three particles ranged from 0.112 to 0.176, indicating an acceptable homogeneity. Table 1 also showed that the zeta potential of G-Z was −41.43 mV and then ramped to −45.93 mV (according to its absolute value, same hereinafter) after zein nanoparticles coated with CMCS. The increase of zeta potential indicated the negative charged CMCS adsorbed on the surface of zein nanoparticles. It was also noted in Table 1 that the zeta potential decreased to −36.57 mV after calcium ions were added into the dispersion system, suggesting that calcium ions were adsorbed on the surface of CMCS or zein molecules, which contributed to cross-linking them into nanoparticles. Strong electrostatic attraction would be generated when the adsorption of Ca^2+^ on CMCS, bringing the CMCS molecules to approaching and then cross-linked these molecules to form spherical nanoparticles.

**Figure 6:**
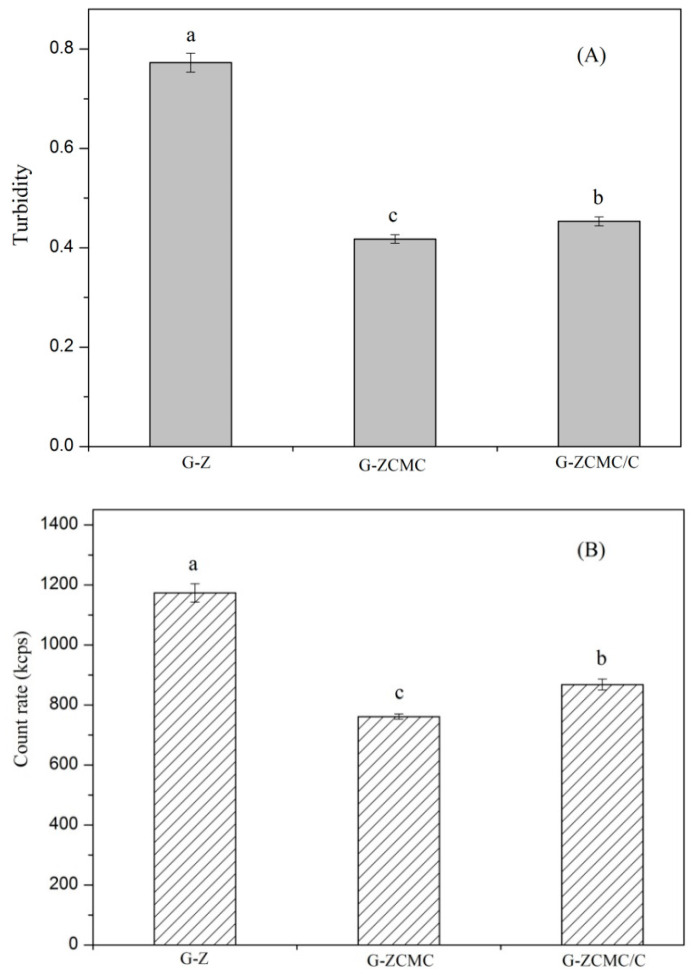
Turbidity (**A**) and count rate (**B**) of different prepared zein nanoparticles. Data are presented as mean ± standard deviation (*n* = 3). Means with different small letters demonstrate significant differences (*p* < 0.05).

The turbidity and count rates of G-Z, G-ZCMC, and G-ZCMC/C dispersions are shown in Figure 6. It can be seen that G-Z showed the highest turbidity, whereas G-ZCMC exhibited the lowest turbidity, which is in contrast with the particle sizes distribution of the studied samples (Table 1). The results are in conflict with what Luo et al. [34] reported. However, the turbidity of the colloidal dispersion for the particle size and other factors, such as particle number in the dispersion, should also be considered. As shown in Figure 6B, G-Z also showed the highest count rate. Weber et al. [35] reported that the count rate of a colloidal system was calculated according to the intensity of the scattered laser, which is proportional to the sixth power of particle sizes as well as the number of the particles. Teng et al. [22] stated that the increase of count rates indicated the increase of nanoparticle numbers if the sizes of colloidal systems were comparable. Therefore, it suggests that G-Z showed the highest particle number. Thus, in the current study, the turbidity of the sample was mainly influenced by the particle number rather than particle size. In addition, Figure 6B shows that calcium ions enhanced the count rate of the colloidal particle, which agreed with previous researches. Teng et al. [22] reported that the addition of calcium (at the low concentration) enhanced the count rate of the soy protein isolate and CMCS complex.

Besides, Table 1 also showed the EE and LE of G-Z, G-ZCMC, and G-ZCMC/C. The EE of G-Z, G-ZCMC, and G-ZCMC/C were 63.32%, 76.15%, and 89.64%, respectively. As shown in Table 1, the LC of G-Z, G-ZCMC, and G-ZCMC/C ranged from 1.82% to 3.65%. The result demonstrated that CMCS complex significantly enhanced the EE of genistein, which could be attributed to the fact that the genistein on the surface of zein nanoparticles was encapsulated after CMCS coating. Several previous researches also reported that CMCS coating significantly enhanced the EE of curcumin in kafirin nanoparticles [17], vitamin D_3_ in zein, and soy protein nanoparticles [19,22]. It was noted that the EE improvement effect of genistein was more pronounced after calcium ion cross-linked with the nanoparticles. Denser coatings would be formed on the surface of zein nanoparticles after the addition of calcium ions to the system, which result from the electrostatic interaction between calcium ions and CMCS, thus enhancing the EE of genistein. 

### 3.2. Morphological Observation

**Figure 7:**
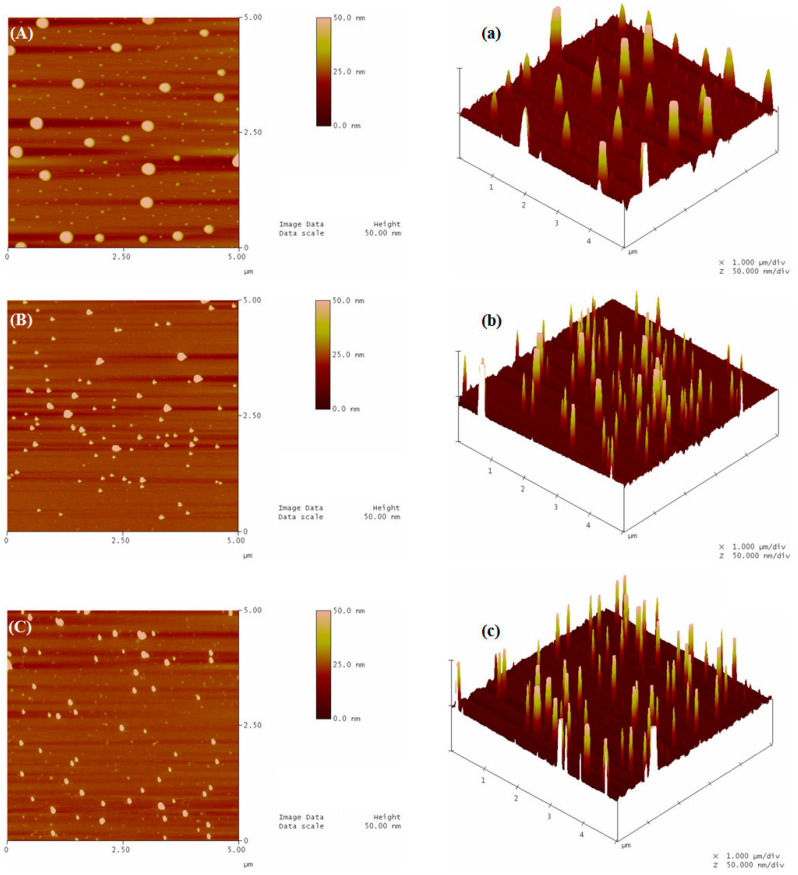
Atomic force microscopy (AFM) images of G-Z (**A**,**a**), G-ZCMC (**B**,**b**), and G-ZCMC/C (**C**,**c**). (**A**–**C**) height image; (**a**–**c**), 3-dimensional image.

The morphological observation of G-Z, G-ZCMC, and G-ZCMC/C nanoparticles were performed by AFM. The representative photographs are shown in Figure 7. It revealed that the liquid–liquid phase separation method employed in this study successfully fabricated genistein encapsulated zein nanoparticles. The G-Z nanoparticles showed a classic spherical shape. In addition, it was noteworthy that the produced G-Z nanoparticles showed a smooth surface without any irregular and rough structures. After decoration with CMCS, the morphology of the zein nanoparticles were altered, and no prefect spherical or disk-shaped particles were observed (Figure 7B). The produced G-ZCMC nanoparticles depicted a roughly round shape with many irregular and rough structures, which might be ascribed to the coating of highly hydrophilic CMCS on the nanoparticles. Early studies stated that the shape of nanoparticles would be generally altered from smooth surfaced ones to rough and irregularly globular shaped ones after coating with a second polymer [36,37,38,39]. Interestingly, the addition of calcium ions made the shape of the particles become spherical and smooth, suggesting the cross-linking effect between calcium ions and CMCS/zein nanoparticles. In addition, it was observed that some relatively large particles were depicted in Figure 7A–C, which could be ascribed to the agglomeration, aggregation, and fusion of some smaller nanoparticles during cast-drying process [40,41,42] before AFM observation. Our speculation agrees with earlier investigations stating that the remarkable aggregation of protein nanoparticles involved with the drying process [22,42]. Zhong and Jin [42] and Luo et al. [19] have stated that zein nanoparticles fabricated with phase separation method were involved in low-energy input techniques and were thus more prone to aggregating, especially when compared with high speed homogenization or high pressure microfluidization techniques. Furthermore, it was also found that coating with CMCS reduced the aggregation of zein nanoparticles for the few amount of big particles observed in Figure 7B, implying that adding CMCS improved the stability of zein nanoparticles against aggregation. This improvement was more remarkable with the addition of calcium ions for cross-linking between CMCS and calcium ions (Figure 7C). In addition, the particle size depicted from AFM photos was smaller than that of our data, which was evaluated via dynamic light scattering (Table 1). The obtained phenomenon was similar to previous studies that reported how drying and staining processes may result in size alteration during conventional AFM measurements, which may not be in accordance with that the result observed using DLS [43]. This difference may be due to the evaporation of water in the nanoparticles after processed by the AFM instrument, thereby resulting in the shrinkage of some nanoparticles. Another reason is that DLS measures the hydrodynamic radius, which is larger than the “real” radius.

### 3.3. FTIR Study on the Nanoparticles

**Figure 8:**
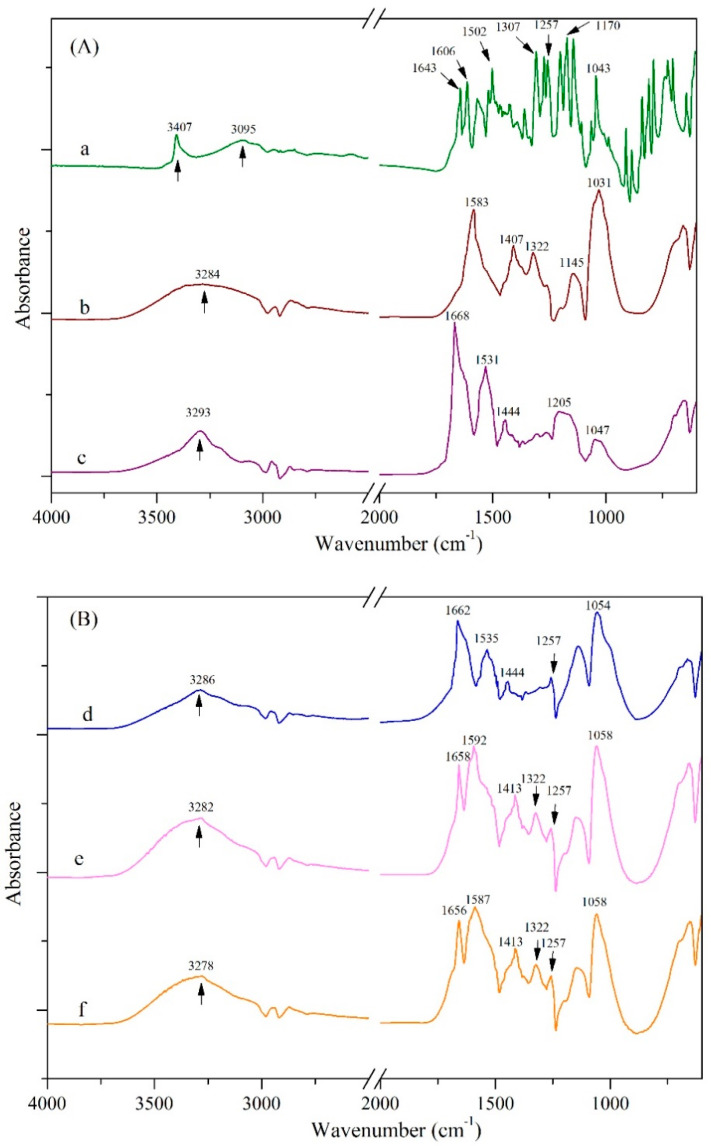
FTIR spectra of the three used pure compounds (**A**) and three fabricated nanoparticles (**B**). **a**, native genistein; **b**, CMCS; **c**, zein; **d**, G-Z nanoparticle; **e**, G-ZCMC nanoparticle; **f**, G-ZCMC/C nanoparticle.

FTIR was conducted to study the intermolecular interactions inside the nanoparticles, and the results are depicted in Figure 8. An interesting characterization peak presented in 3200−3500 cm^−1^ was found in the infrared spectra, suggesting hydrogen bonding [19]. In the original spectra of genistein, CMCS, and zein, the characteristic bands of hydrogen bonds were distributed at 3407, 3284, and 3293 cm^−1^, respectively. The two bands of zein spectra at 1668 cm^−1^ and 1531 cm^−1^ demonstrated amide I band (C=O stretching) and amide II band (C-N stretching), respectively [19]. For native genistein, the apparent peaks were distributed at 3407 cm^−1^ and 3095 cm^−1^, which could be attributed to the stretching vibrations of O-H and aromatic C–H, respectively. The C–O, C–C, C–O–C, and C–C stretching vibrations of genistein were presented at 1643, 1606, 1307–1150, and 1260–1000 cm^−1^, respectively [44]. For the infrared spectra of CMCS, the characteristic peaks were appeared at 1583 cm^−1^, 1407 cm^−1^, and 1031 cm^−1^, which are assigned to C=O, CH_2_COO–, and –C–O– stretching vibrations [17,24,45]. After the formation of G-Z, G-ZCMC, and G-ZCMC/C nanoparticles (Figure 8B), it was found that most characteristic peaks of genistein were disappeared or overlapped by absorption peaks of a biopolymer matrix, indicating the successful encapsulation of genistein. It was noteworthy that every formulated nanoparticle exhibited a prominent absorption peak of genistein at 1257 cm^−1^ in the infrared spectra, implying the existence of genistein. Particularly after the formation of particles, it was noted that hydrogen bands of genistein at 3407 cm^−1^ were disappeared and merged to 3286, 3282, and 3278 cm^−1^ in the spectra of G-Z, G-ZCMC, and G-ZCMC/C. This implied that strong hydrogen bonds formed among genistein, zein, and CMCS. This observed phenomenon might be due to the reason that hydrogen bonds can form between hydroxyl groups of genistein and amide groups of glutamine in zein. Additionally, both amide groups and hydroxyl groups are presented in the structure of CMCS; hence, the formation of hydrogen bonds would be easily occurred between CMCS and genistein, as well as CMCS and zein. Thereby, the hydrogen bonds among zein, genistein, and CMCS were regarded as one of the predominant forces that facilitated the formation of nanoparticle.

Furthermore, Figure 8 shows that amide I and amide II bands of zein were shifted from 1668 to 1662 cm^−1^ and from 1531 to 1535 cm^−1^, respectively, after G-Z particle formation. This result can be ascribed to hydrogen bonds forming between the carbonyl group in amide bond and the phenolic hydroxyl in genistein during G-Z nanoparticle fabrication. Amide I and II bonds shifted more apparently in G-ZCMC and G-ZCMC/C, likely because of the formation of additional hydrogen bonding between carbonyl group in amide bond and carboxylic hydroxy group in CMCS. Additionally, hydrophobic attraction can exist during the formation of nanoparticles since genistein and zein are both hydrophobic compounds [17]. Therefore, the hydrophobic effect was considered to be another force for the formation of nanoparticles to entrap genistein.

### 3.4. Proposed Mechanism for the Formation of Genistein Encapsulated Nanoparticles

**Figure 9:**
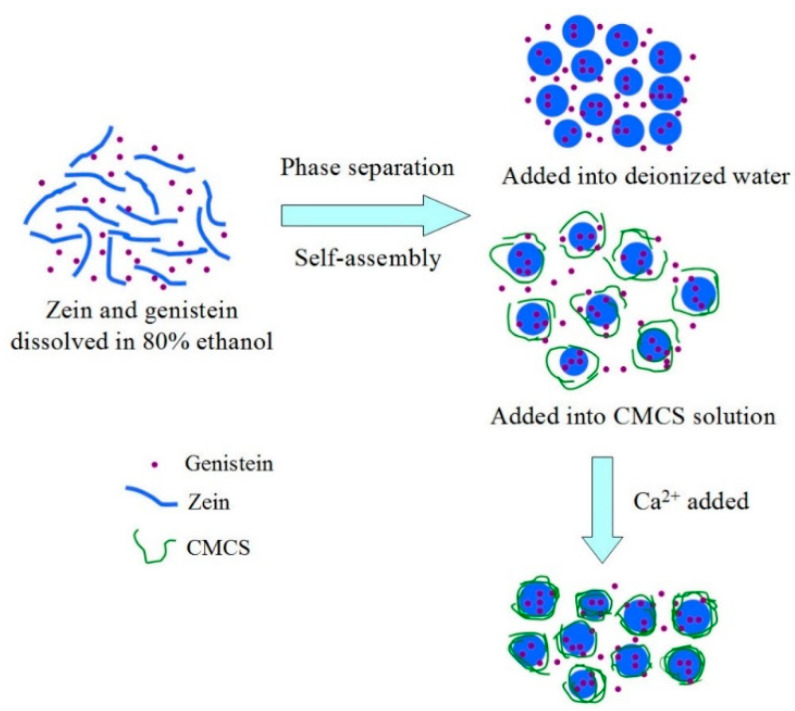
Schematic illustration of the formation mechanism of zein/carboxymethyl chitosan complex for encapsulation of genistein.

In light of the observed results, the mechanism for the formation of genistein encapsulated G-Z, G-ZCMC, and G-ZCMC/C nanoparticles is illustrated and presented in Figure 9. When a genistein ethanol aqueous solution was dropped into a zein ethanol aqueous solution, genistein was wrapped by zein molecules and a thin film formed on the surface via hydrophobic attraction and hydrogen bonds forces between the two molecules. After genistein and zein stock solution were dispersed into bulk water, the polarity of the solvent system remarkably increased, and ethanol in the droplets migrated into the bulk water because of the great miscibility of water and ethanol. As a consequence, the zein became insoluble, resulting in its self-assembling into nanoparticles with co-dissolved genistein mainly via hydrogen bonding and hydrophobic interaction. As soon as the above obtained zein nanoparticle dispersions added into CMCS solution, it was likely that the surface of zein particles was adsorbed with CMCS mainly ascribed to the action of hydrophobic interaction and electrostatic attraction. CMCS can be considered as an amphiphilic compound that the carbon bone of glucose can serve as hydrophobic group, whereas NH_2_ can be used as a polar head. During the formation process of CMCS complexed nanoparticles, NH_2_ and carboxylic groups of CMCS served as the outer hydrophilic shell, whereas the carbon bone of glucose formed the inner hydrophobic domains. As soon as calcium ions were introduced into the colloidal particle system, the negatively charged G-ZCMC nanoparticles would be attracted by the positively charged calcium ions through electrostatic interactions. As a result, the surface of CMCS or zein molecules were adsorbed via calcium ions that brought the CMCS molecules to approach and tightly cross-linked these molecules to form spherical nanoparticles. Thus, the sizes of the formed nanoparticles became smaller and more genistein was encapsulated in the nanoparticles (Table 1).

### 3.5. Effect of CMCS Addition on the Thermal and Storage Stability of Zein Nanoparticles

#### 3.5.1. Influence of Heating Temperature on Particle Stability

**Figure 10:**
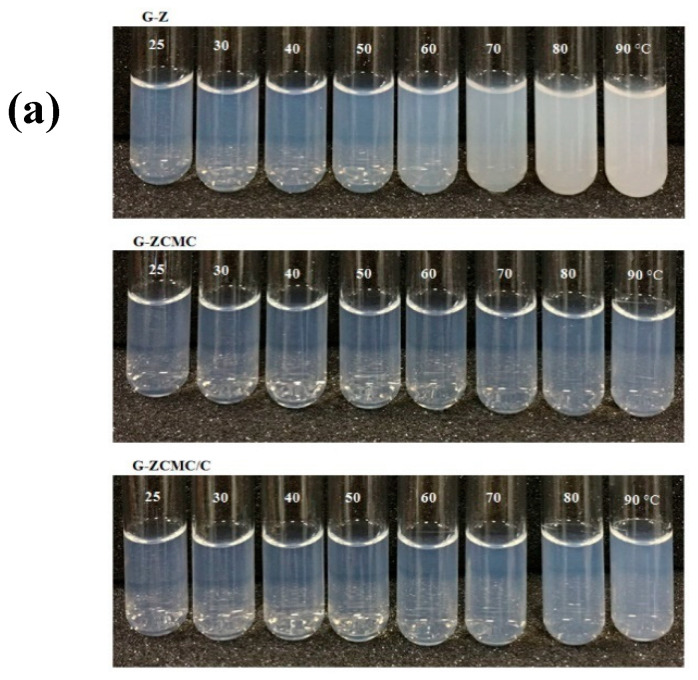

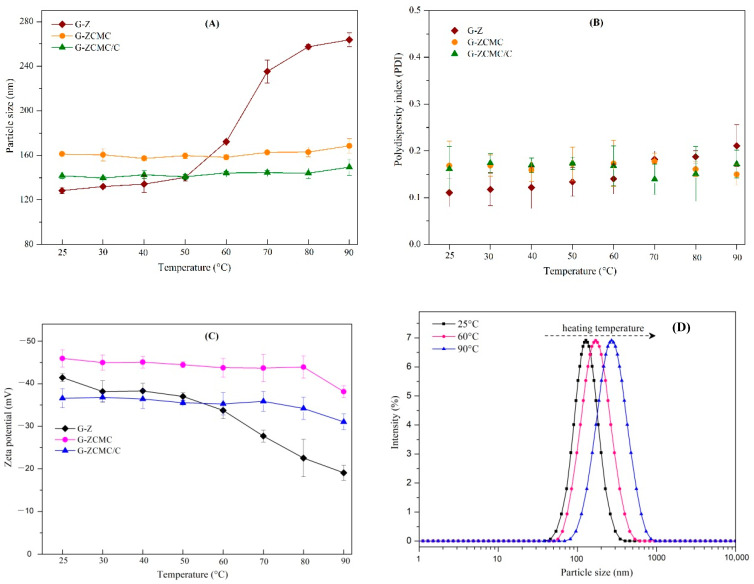
(**a**) Effect of different heating temperature for 30 min on macroscale stability of uncoated CMCS and CMC/C coated zein nanoparticles. Change of particle diameter, (**A**) PDI (**B**), and zeta potential (**C**) of G-Z, G-ZCMC, and G-ZCMC/C nanoparticles to different temperature heating for 30 min. (**D**) Particle size distribution of G-Z heated for 30 min at 60 °C and 90 °C, respectively. Data are presented as mean ± standard deviation (*n* = 3).

The nanoparticle delivery systems involved in the production of commercial food may undergo a range of thermal processes. The heat treatment used in the food process may range from relatively low temperatures to high ones [46]. In order to effectively deliver genistein, the integrity of biopolymer particles need to be maintained during these heat treatment processes. Therefore, the effect of different thermal treatments on the attributes of formed zein nanoparticles was evaluated. The freshly prepared G-Z, G-ZCMC, and G-ZCMC/C was treated for 30 min with various temperatures ranging from 30 to 90 °C; the result is shown in Figure 10. It was observed that G-Z particles were quickly destabilized after the heated temperatures exceeded 60 °C, as revealed by an apparent increase in solution turbidity and mean particle size (Figure 10a,A). This observed result can be ascribed to the reason that more zein protein molecules unfolded and became reactive at higher temperatures, and the collision frequency between protein and protein also increased when the temperature increased [46]. In addition, the reduction of electrical charges on the zein nanoparticles (Figure 10C) resulted in the reduction of electrostatic repulsion between the particles, which led to the increased diameter size of the nanoparticles. Moreover, relatively low electrical charge was reported for inducing Ostwald ripening or coalescence after thermal treatment, which was another reason for the increase of particle size [36]. However, the PDI values of each studied sample was lower than 0.3 (Figure 10B), implying that every nanoparticle was still homogeneously distributed in the system after thermal treatment. Coating with CMCS hydrophilic copolymers greatly improved the stability of short-term thermal treatments for prepared nanoparticles, as revealed in Figure 10. G-ZCMC and G-ZCMC/C particle still appeared visibly stable at higher temperature treatments and the aggregation of the studied samples did not occur (Figure 10a). There was no significant alteration of mean particle size and zeta potential when G-ZCMC and G-ZCMC/C were exposed to higher temperatures (Figure 10A). The negative charge of the particles significantly increased after CMCS coating. A highly hydrated polymeric was formed around the particles, contributing to the increase of electrostatic and steric repulsion between the particles [36]. Therefore, the probable reason of the observed result was that coating with CMCS increased the steric and electrostatic repulsion between the formed biopolymer particles, which contributed to the reduction of heat-induced particle aggregation. More investigations are required to fully demonstrate the nature of the stabilization mechanism. The obtained result is important as it could provide useful information for the coated zein nanoparticles in industrial food production and in meal preparation that requires heating temperature environments.

#### 3.5.2. Influence of Heating Time on Particle Stability

**Figure 11:**
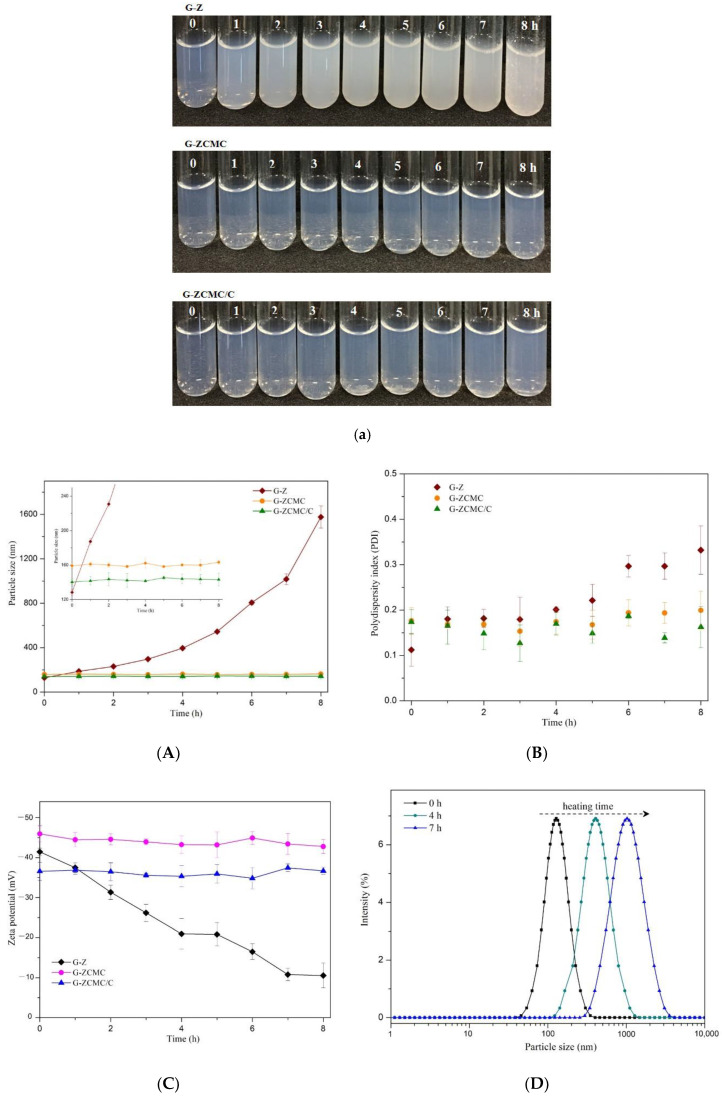
(**a**) Effect of different heating time at 65 °C on macroscale stability of G-Z, G-ZCMC, and G-ZCMC/C nanoparticles. Particle attributes alternation of particle diameter (**A**), PDI (**B**), and zeta potential (**C**) of G-Z, G-ZCMC, and G-ZCMC/C nanoparticles to different heating time at 65 °C. (**D**) The particle size distribution of G-Z heated at 65 °C for 4 h and 7 h, respectively. Data are presented as mean ± standard deviation (*n* = 3).

The G-Z, G-ZCMC, and G-ZCMC/C particle dispersions were also heated for different times at 65 °C and we studied the effect on particle attributes, i.e., particle size, PDI, and zeta potential (Figure 11). The solution turbidity and particle size of G-Z particle dispersions increased gradually, whereas its zeta potential value (in terms of absolute value) decreased under heating time, implying the occurrence of protein aggregation during this heating period. Previous studies also reported that heating significantly reduced the zeta potential value and increased the particle size of the studied samples [47]. In addition, extensive aggregation of zein particles occurred after heating for 8 h, which was revealed by visual particle sedimentation and a remarkable particle size increase (Figure 11). The apparent occurrence of particle aggregation over this heating time was likely caused because thermal treatment reduced the particles’ electrical charge, which led to a weak electrostatic repulsive force between particles. Furthermore, collision frequency typically increased between zein particles during heating treatment, and resulted in proteins unfolding and exposing themselves to hydrophobic groups, were responsible for particle aggregation induced by heating treatment. 

No significant changes were observed for particle size, zeta potential, PDI, and turbidity of G-ZCMC and G-ZCMC/C under different heating times compared to the sample before the heating test. This indicates that there was no change of these particle attributes (Figure 11A–C), i.e., G-ZCMC and G-ZCMC/C were stable and homogeneously distributed in the dispersions. Obviously, CMCS coating enhanced the thermal stability of the zein nanoparticles, which was probably attributed to the increased forces of electrostatic and steric repulsion after CMCS coating on particles. 

#### 3.5.3. Influence of Storage on Particle Stability

**Figure 12:**
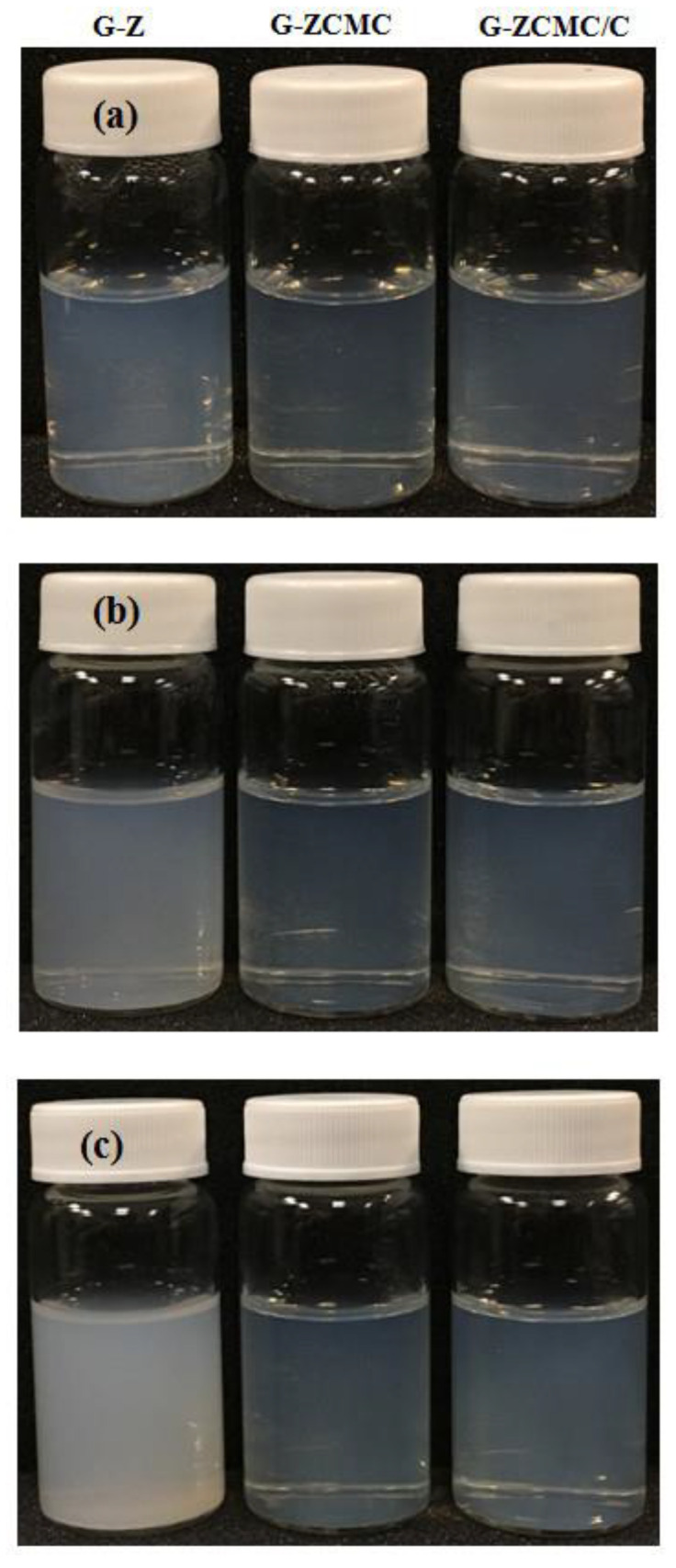
The images of G-Z, G-ZCMC, and G-ZCMC/C before (**a**) and after 60 days of storage at 4 °C (**b**) and 25 °C (**c**), respectively.

**Figure 13:**
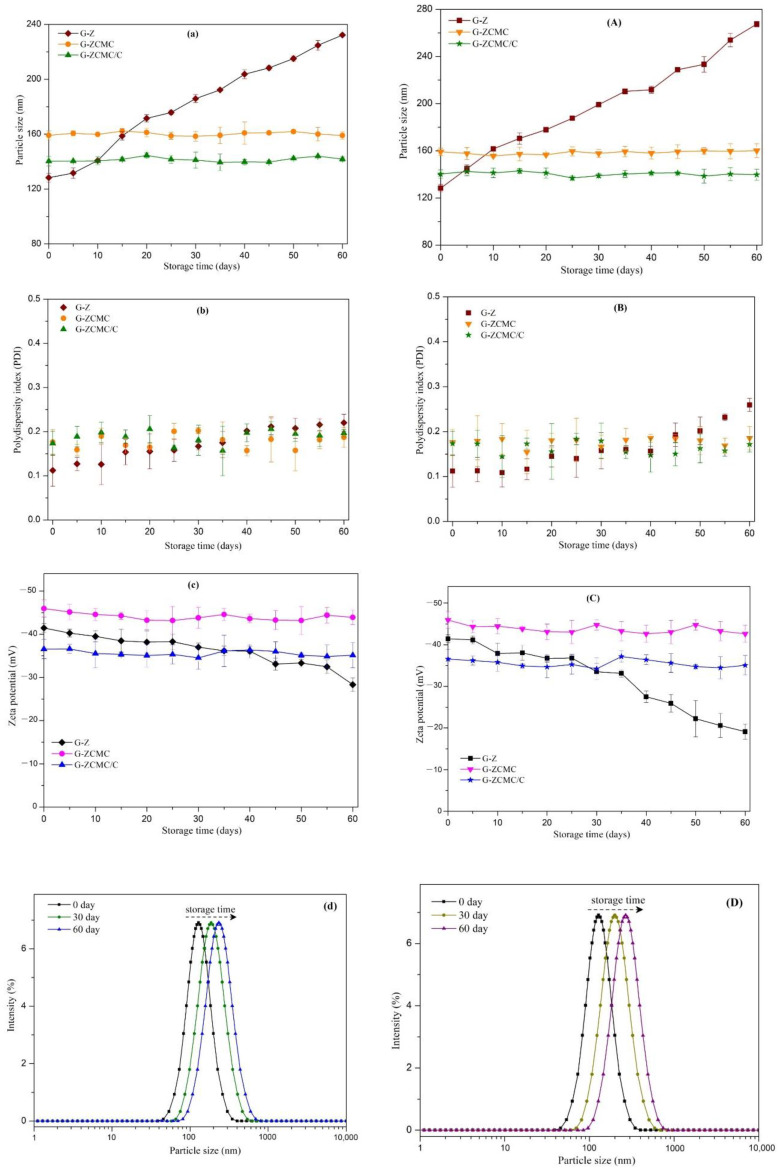
Change in particle size (nm), polydispersity index (PDI), and zeta potential of G-Z, G-ZCMCS, and G-ZCMC/C nanoparticles at 4 °C (**a**–**c**) and 25 °C (**A**–**C**). The size distribution of G-Z stored for 60 days at 4 °C (**d**) and 25 °C (**D**), respectively. Data are presented as mean ± standard deviation (*n* = 3).

To develop an excellent functional beverage formulation, one of the critical quality characteristics is the product’s storage stability, which greatly influences the industrialization of the final product [46]. The storage stability of freshly fabricated nanoparticle formulations was assessed at two common different storage conditions (i.e., 25 °C and 4 °C) for two months; the results are depicted in Figure 12 and Figure 13. It was noted that the particle size of G-Z continuously increased when stored at room temperature and refrigeration conditions for two months; however, no macroscale suspension instability appeared. The PDI values of every studied G-Z sample at different storage conditions were still at a low level (<0.3), suggesting that they maintained a unified distribution. The zeta potential was relatively stable when G-Z formulations were stored at refrigeration conditions (Figure 13c). The addition of CMCS significantly improved the storage stability of zein nanoparticles, as demonstrated in Figure 12 and Figure 13. Both G-ZCMC and G-ZCMC/C formulations were quite stable and attributes such as particle size, zeta potential, and PDI were well maintained when stored in two different storage conditions (Figure 13a–c,A–C). Previous investigations have stated that the stability of particle interfacial structure is better after coating with the protein/polysaccharide complex compared to the protein alone, because it ascribes to the formulated stronger force of the hydrophobic interaction, as well as the electrostatic hydrogen and steric repulsion between the two polymers [16,21,48].

### 3.6. Evaluation of Antioxidant Activity 

**Figure 14:**
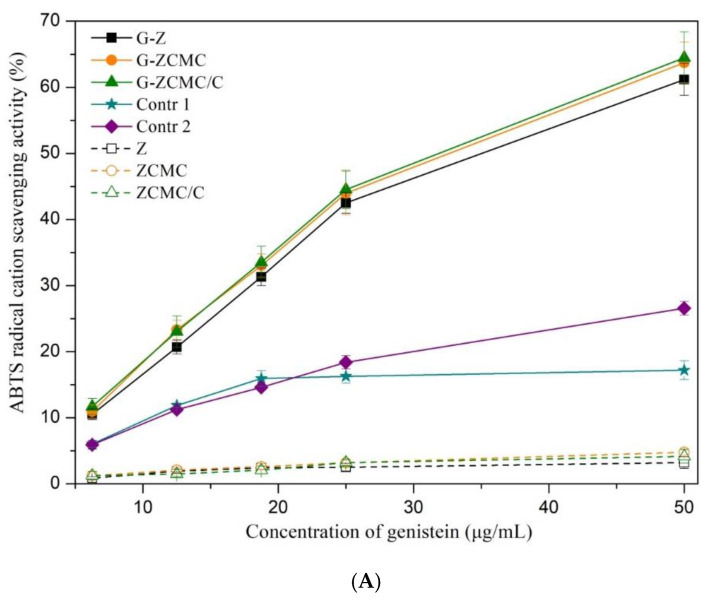

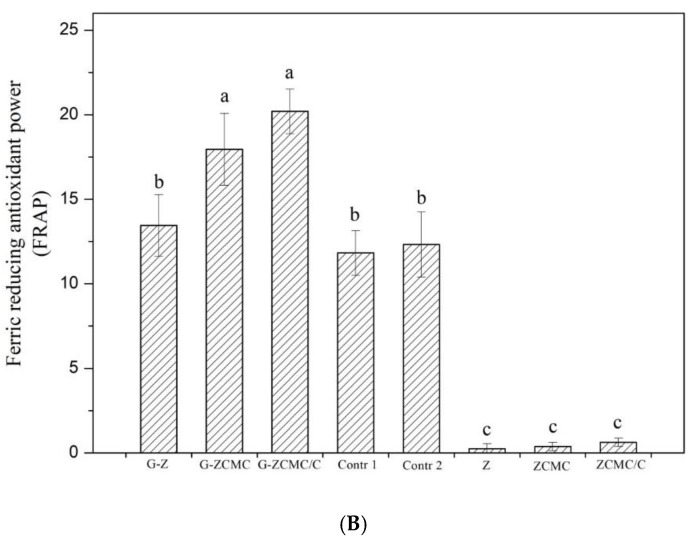
ABTS radical cation scavenging activity (**A**) and ferric reducing antioxidant power (**B**) of genistein-loaded zein nanoparticles. Two controls were conducted in parallel for comparison, including genistein pre-dissolved in ethanol as stock solution (1.2 mg/mL) and then diluted respectively with water (Control 1) and 80% ethanol (Control 2). Data are presented as mean ± standard deviation (*n* = 3). Means with different small letters demonstrate significant differences (*p* < 0.05).

Genistein is an isoflavone reported to possess strong biological activity, such as antioxidant activity. However, it was reported in several previous studies that genistein exhibited weak antioxidant capacity in the aqueous phase due to its poor water solubility (less than 1 µg/mL), which results in its low bioavailability and in vivo efficacy, thereby limiting its application in food products [49]. Thus, it is extremely necessary to improve its water dispersibility and biological activity. The ABTS·^+^ scavenging activity and ferric reducing antioxidant power (FRAP) of genistein before and after encapsulation are depicted in Figure 14. At 50 µg/mL of genistein, the ABTS^+^ scavenging activity of the three genistein-loaded nanoparticles were about 60%, whereas the values for free genistein dispersed in water (Control 1) or dissolved in ethanol (Control 2) were only 17.19 and 26.57%, respectively (Figure 14A). Obviously, all three genistein-loaded nanoparticles showed a significantly higher ABTS^+^ scavenging activity when compared to free genistein dispersed in water or dissolved in ethanol. This effect was in a dose–response dependent manner as the genistein concentration increased. Genistein could be well dispersed in the reaction aqueous medium after being encapsulated by zein nanoparticles. Moreover, genistein-loaded nanoparticles exhibited big surface areas, which together facilitated the kinetics reaction of genistein with ABTS^+^ in the aqueous environment [15,28]. In contrast, free genistein could not be well distributed in aqueous media and thereby most of genistein separating out, which led to a reduced contact probability between genistein and ABTS^+^, and consequently lower antioxidant activity. Several previous publications also found that encapsulation of other poor water solubility phenolics such as curcumin in zein or casein colloidal particles could remarkably improve its ABTS^+^ scavenging activity in the aqueous phase [28,50,51]. No significant differences were found among different genistein encapsulated zein nanoparticles. In addition, the ABTS^+^ scavenging activity of three empty (genistein-free) nanoparticles equivalent to the concentrations of genistein were also measured, but this was found to be very weak (Figure 14A). The ABTS·^+^ scavenging ability of the three empty nanoparticles at the corresponding concentration of genistein (50 μg/mL) nanocomplex were found to below 5%, which clearly demonstrated that the improved antioxidant activity of genistein was ascribed to the encapsulation process, rather than the presence of zein/CMCS.

The FRAP of free genistein (unencapsulated) and genistein encapsulated in nanoparticles were also determined, and the results are shown in Figure 14B. It was found that genistein-loaded zein nanoparticles coated by CMCS significantly improved the FRAP compared to free genistein, which mainly improved water dispersibility and the large surface area of G-ZCMC and G-ZCMC/C nanoparticles. However, it was noteworthy that the FRAP of genistein was not remarkably (*p* > 0.05) enhanced when encapsulated in zein nanoparticles, which might be due to the reason that G-Z nanoparticles were unstable in acid conditions (pH 3.6 for testing FRAP), causing the aggregation of G-Z nanoparticles and restricting the amount of genistein available to interact with Fe^3+^ in the aqueous phase. In addition, three empty (genistein-free) nanoparticles showed negligible FRAP tested in this study (Figure 14B). This observed result implied that the enhanced FRAP of the genistein-loaded G-ZCMC and G-ZCMC/C nanoparticles are primarily due to the encapsulation of genistein rather than the presence of zein/CMCS.

### 3.7. Controlled Release of Genistein from Z-G, Z-GCMC, and Z-GCMC/C

**Figure 15:**
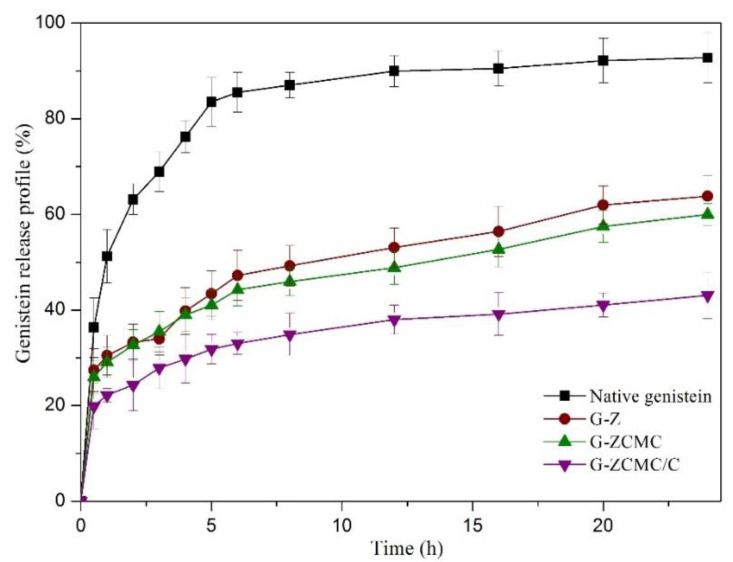
The genistein release profile of native genistein, G-Z, G-ZCMC, and G-ZCMC/C nanoparticles. Data are presented as mean ± standard deviation (*n* = 3).

The fabricated particles were studied to assess the release profile of genistein. A dialysis bag with a 12 kDa cut-off was conducted in this study in order to avoid nanoparticles escaping into the release medium. Therefore, only free released genistein was evaluated in this assay. As denoted in Figure 15, a quick diffusion rate was found for unencapsulated genistein (free genistein), with above 50% being observed in the first 1 h and reaching about 85% after 5 h of release. When free genistein was exposed to the release medium and not encapsulated in any biopolymers, it diffused quickly through the dialysis membrane. In contrast, a much slower kinetic release profile of genistein was observed during the entire release process when genistein was encapsulated in a zein/CMCS biopolymer matrix (Figure 15). All of the three nanoparticle formulations (G-Z, G-ZCMC, and G-ZCMC/C) exhibited similar release trends. Biphasic release patterns were detected and an early rapid release occurred at 0.5 h. Then, a sustained genistein release was observed for more than 24 h. This result was consistent with Shaikh et al. [32], who employed the same method to study the release profile of curcumin in nanoparticles. The genistein delivery system carried out in the present study was regarded as the fabricated nanoparticles with hydrophilic shell and hydrophobic core, which has been broadly investigated in previous studies for encapsulating hydrophobic biological compounds via hydrophobic interactions [17,52]. The sustained release of hydrophobic nutraceuticals from polymeric nanoparticles has been reported in previous studies by erosion of the polymer or by diffusion of hydrophobic compounds from the polymeric nanoparticles [12]. It is worth mentioning that, typically, biphasic release patterns of genistein were observed for G-Z, G-ZCMC, and G-ZCMC/C formulations. The early burst release was mainly ascribed to the genistein located on the periphery of the polymer and poorly entrapped in the nanoparticle core, which was easily diffused into the release medium. The following continuous release was mainly observed via nanoparticle degradation and genistein diffusion, which was well encapsulated in the polymer core [32].

In addition, Figure 15 also shows that G-Z and G-ZCMC nanoparticles exhibited similar release results, both of which 60% genistein was released after 24 h. However, it was noted that only 43.08% of the total genistein was released in 24 h for G-ZCMC/C. According to the above results, it could be concluded that encapsulated nanoparticles controlled the release of genistein in the studied release medium. CMCS coating and calcium ions cross-linking improved the controlled release effect. Denser CMCS coatings would be formed on the surface of zein nanoparticles after addition of the cross-linker calcium ions, thereby contributing to the controlled release. It was well documented in the literature that various biopolymer coatings enhance the controlled releases of compounds from nanoparticles [19,22,53]. 

### 3.8. Photochemical Stability Against UV light

**Figure 16:**
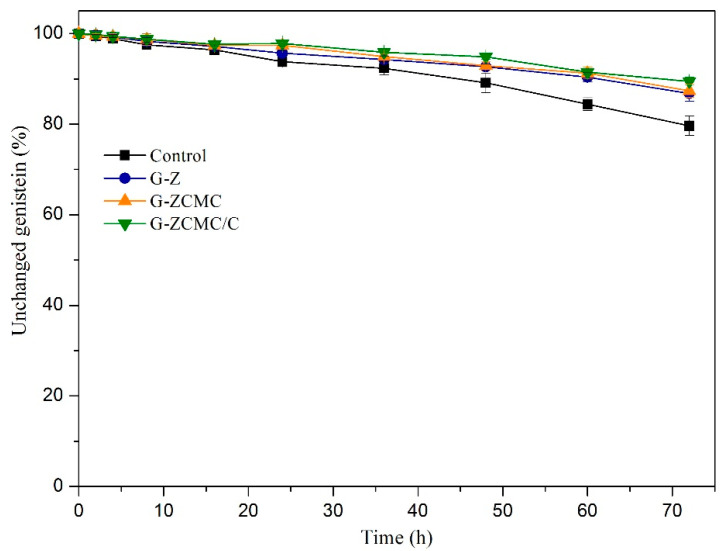
Percentage of unchanged genistein during UV irradiation as a function of time for native genistein, G-Z, G-ZCMC, and G-ZCMC/C suspensions. Data are presented as mean ± standard deviation (*n* = 3).

UV irradiation is an effective and economic sterilization approach during the production of food and beverage [36]. However, light has been reported to be a major factor leading to isomerization, oligomerization, and oxidation of phenolic compounds and flavonoids [52], resulting in their reduction of biological activity. Genistein exhibited UV absorption ability for its structure possesses aromatic rings, which might cause genistein instability, oxidation, or degradation when exposed to UV light. In the present study, the effect of encapsulation on genistein’s photo-stabilities was studied and the results are denoted in Figure 16. No significant difference was observed among the native genistein and encapsulated genistein samples when exposed to UV treatment for 36 h. However, it was found that encapsulation in zein nanoparticles protected genistein against UV light when treatted for 72 h, and the CMCS coating slightly improved the protection effect for genistein. Among the different samples studied, G-ZCMC/C nanoparticles showed the greatest protection for genistein, in which 89.46% of total genistein was not influenced by UV irradiation. Therefore, genistein encapsulated in zein colloidal particles enhanced the UV irradiation stability of genistein when compared to native genistein. Xiao et al. [17] reported that kafirin/CMCS nanoparticles provided protection for curcumin against UV treatment, and they attributed the enhanced UV stability to the barrier effect of biopolymer. Double bonds and aromatic side groups were included in the structure of zein protein, which can absorb UV light when protein is exposed to UV irradiation [19]. Therefore, this contributed to the protective effect of genistein against UV light. Even better protection was observed by CMCS coating and calcium ions cross-linking was mainly ascribed to the formed thicker and denser physical barrier. Therefore, encapsulation of genistein into polymeric matrix is an effective approach to provide protection for genistein against oxidation and degradation in harsh environment.

## 4. Conclusions

In the present study, genistein-loaded G-Z, G-ZCMC, and G-ZCMC/C nanoparticles were successfully prepared using a liquid−liquid phase separation method. Several techniques, including UV-vis, DLS, FTIR, and AFM were carried out to characterize and identity the physicochemical properties of the genistein-loaded nanoparticles. Genistein successfully encapsulated in nanoparticles were mainly driven by hydrophobic interaction, hydrogen bonding, and electrostatic attraction. G-ZCMC/C exhibited the highest genistein encapsulation efficiency among the three nanocomplexes. Hardening with CMCS greatly improved the thermal and storage stability of zein nanoparticles. Furthermore, encapsulation of genistein in G-Z, G-ZCMC, and G-ZCMC/C nanoparticles significantly improved its antioxidant capacity in aqueous environment for its increased water dispersibility. The photochemical stability against UV light of genistein was greatly enhanced after encapsulated in G-Z, G-ZCMC, and G-ZCMC/C nanoparticles. G-ZCMC/C showed the best protection due to the thicker and denser physical polymer matrix. All three nanoparticle formulations provided a controlled release of genistein, while G-ZCMC/C showed the best performance. Therefore, this study demonstrated that zein/CMCS complex nanoparticles system is a promising delivery vehicle to promote genistein application in functional food, pharmaceutical, and cosmetic industries. Further investigations should concentrate on evaluating the cytotoxicity, cellular uptake ability, and in vivo biological activity of genistein-loaded nanocomplexes.

## Figures and Tables

**Table 1:** Mean particle size, polydispersity index (PDI), zeta potential (mV), encapsulation efficiency, and loading efficiency of G-Z, G-ZCMC, and G-ZCMC/C.

Samples	Particle Size (nm)	PDI	Zeta Potential (mV)	EE (%)	LE (%)
G-Z	128.33 ± 3.09 ^c^	0.112 ± 0.036 ^a^	−41.43 ± 0.95 ^b^	63.32 ± 5.03 ^c^	3.65 ± 0.20 ^a^
G-ZCMC	159.20 ± 3.18 ^a^	0.176 ± 0.029 ^a^	−45.93 ± 2.00 ^a^	76.15 ± 2.61 ^b^	1.82 ± 0.13 ^b^
G-ZCMC/C	140.37 ± 3.39 ^b^	0.174 ± 0.027 ^a^	−36.57 ± 2.25 ^c^	89.64 ± 4.04 ^a^	1.98 ± 0.33 ^b^

Each value was expressed as mean ± standard deviation (*n* = 3). Means with different small letters within a column demonstrate significant differences (*p* < 0.05).

## Abbreviation

CMCS	carboxymethyl chitosan
G-Z	genistein-loaded zein nanoparticles
G-ZCMC	genistein-loaded zein nanoparticles coated with CMCS
G-ZCMC/C	genistein-loaded zein nanoparticles coated with CMCS and crosslink with calcium ions
DLS	dynamic light scattering
AFM	atomic force microscopy
FTIR	Fourier transform infrared spectroscopy.
